# Translating person-centered care into homecare practice: challenges and innovations in implementing dementia policy in Sweden

**DOI:** 10.3389/frdem.2026.1814577

**Published:** 2026-05-15

**Authors:** Andrea Nakakawa, Jenny Löyttynen, Daniela Sangiorgi, Lena Marmstål Hammar

**Affiliations:** 1 Design Department, Politecnico di Milano, Milan, Italy; 2 Faculty of Engineering and Health Sciences, Mälardalen University, Västerås, Sweden

**Keywords:** dementia, home care, participatory design, person-centered care, policy enactment

## Abstract

**Introduction:**

Despite strong commitments, translating person-centered and dementia care principles into practice remains challenging in homecare settings. In Sweden, national and regional frameworks such as Nära Vård (Good and Close Care) and the Sörmland Dementia Strategy promote autonomy, participation, and collaboration. Yet, their enactment unfolds within layered institutional systems characterized by multiple logics, documentation demands, and fragmented responsibilities, which often dilute the intent of person-centered policies. The study applied a policy enactment perspective to examine how person-centered and dementia-related policies are interpreted, negotiated, and translated into municipal homecare services. It further explores how participatory, design-driven methods can support policy enactment by providing structured arenas for collective sensemaking and resource negotiation across system levels.

**Methods:**

A qualitative design was employed in the county of Sörmland, Sweden, combining 17 semi-structured interviews with policymakers, municipal managers, and homecare staff, and two participatory workshops (*n* = 14–18) involving actors ranging from frontline staff to managerial levels. Interview data was analyzed using inductive and abductive thematic analysis, while workshop data were examined using the framework method to identify shared interpretations and enactment conditions. Patient and Public Involvement (PPI) activities with people living with dementia and unpaid carers informed the design and validated the findings.

**Results:**

Findings show that policy enactment is constrained by fragmented organizational structures, limited prioritization of dementia, uneven (dementia-specific) knowledge across roles, workforce instability, time pressures, and weak feedback mechanisms. Documentation-heavy routines often reinforce biomedical notions of “good care,” limiting the mobilization of relational, person- centered practices. Participatory workshops facilitated the development of provisional, middle-level interpretations that can help bridge abstract policy ideals and situated care practices.

**Discussion:**

Our study contributes by extending policy enactment frameworks into dementia homecare, and by illustrating how participatory approaches can strengthen bottom-up translation mechanisms within complex public care systems.

## 1 Introduction

Despite decades of international commitment, the implementation of person-centered care (PCC) remains a persistent challenge, particularly in dementia services. PCC is commonly grounded in the recognition of personhood, dignity, and agency, emphasizing that individuals living with dementia should be supported as persons rather than defined by diagnosis or functional decline (Kitwood, 1997, 2013). However, translating these values into everyday practice has proven difficult within health and social care systems characterized by multiple professional groups, fragmented responsibilities, regulatory demands, workforce diversity, and resource constraints (Erlandsson and Szebehely, 2024). In such systems, PCC becomes a complex intervention requiring cultural, relational, and organizational change rather than a simple adoption of new practices or tools (McCormack et al., 2010).

These challenges are particularly pronounced in dementia care. While the core principles of PCC remain consistent across settings, their enactment in dementia care involves distinct challenges linked to cognitive decline, communication changes, and increasing dependence (Manthorpe and Samsi, 2016; Nandimath, 2021). Supporting autonomy, participation, and preferences often requires ongoing interpretation, relational work, the involvement of family and informal carers as partners, and the interpretation of non-verbal or evolving expressions of need (Hedman et al., 2022; Xu et al., 2024). As a result, PCC in dementia care is not only about shared decision-making, but also about sustaining personhood and agency under conditions where these may be difficult to articulate or observe (Kitwood, 1997; Brooker, 2007; Hoel et al., 2021). These specificities make the translation of PCC into homecare particularly complex.

Previous research shows that efforts to implement PCC, particularly in dementia settings, often depend on facilitated, participatory, and reflective processes that address not only care practices but also the cultures and contexts in which care is delivered (Manley and McCormack, 2003; Brooker, 2007; McCormack et al., 2010; Marulappa et al., 2022). This suggests that implementation difficulties do not stem solely from a lack of knowledge or resistance to change, but from tensions between policy ideals and the realities of everyday work in complex care environments.

To examine these tensions, this study adopts a policy enactment perspective. Policy enactment refers to the process through which policy intentions are interpreted, negotiated, and translated into everyday practice (Braun et al., 2010), and offers a useful perspective for understanding how policies materialize in real-world contexts (Brower, 2025). Enactment perspectives view policy as something whose meaning is dynamic and continuously produced through sensemaking and translation across organizational levels (Ball et al., 2011; Clarke et al., 2015). This is particularly relevant in dementia care, where policy ideals such as participation, autonomy, and person-centeredness must be continuously re-interpreted in relation to fluctuating cognitive capacity, relational care work, and everyday constraints (Hoel et al., 2021; Hedman et al., 2022; Marulappa et al., 2022).

Sweden offers an interesting context for examining these processes. National and regional reforms strongly endorse person-centered, integrated, and preventive approaches to support aging in place (e.g., National Dementia Strategy 2025–2028; Good and Close Care, 2018; Good and Close Care in Sörmland, 2025), while responsibility for implementation largely rests with municipalities (Agerholm et al., 2024). Despite strong policy ambitions, translating these ideals into daily practice remains a challenge, with persistent gaps in dementia knowledge, language skills, and flexibility in care delivery (Myndigheten för vård- och omsorgsanalys, 2025; Socialstyrelsen, 2024).

While policy enactment theory helps explain how policies are interpreted and adapted across multiple layers, it says less about how actors can collectively bridge interpretive gaps in practice. Here, participatory design-based approaches offer a valuable complement. Participatory design methods and systems thinking can help those involved in care delivery to co-construct meanings, surface tensions between policy intentions and daily realities, and support collective sensemaking across professional and institutional boundaries (Eneberg, 2012; Van Der Velden and Mörtberg, 2014; Jones, 2014, 2018; Matos-Castaño et al., 2020). In this way, systems-aware participatory processes may support more bottom-up forms of policy formulation and implementation (Marchal et al., 2021; Blomkamp, 2022).

The aim of this study is to identify conditions influencing the enactment of person-centered and dementia-related policies in Sörmland's municipal homecare and to explore how participatory, design-driven methods can support these processes. While the empirical material captures a range of experiences, the analysis presented here emphasizes the conditions that constrain enactment, as these provide greater explanatory insight into variation in practice. The study addresses two research questions: (1) What conditions influence the enactment of person-centered care practices in dementia homecare? and (2) How can participatory design-driven methods support such enactment processes?

This study contributes by extending enactment frameworks into the dementia homecare domain, and by examining how participatory approaches may support the local translation of PCC principles in a complex care system.

## 2 Theoretical framework

This study examines how person-centered dementia policies are translated into practice within municipal homecare. It uses policy enactment as the primary analytical lens and draws selectively on sensemaking and socio-material perspectives to explain how policy is interpreted, negotiated, and organized in everyday care. A systems-aware participatory design perspective is then introduced to explore how these processes may be supported in practice.

### 2.1 Policy enactment

We conceptualize policy enactment as the situated and collective process through which policy intentions are interpreted, negotiated, and made meaningful in everyday practice. From this perspective, policies are not implemented as fixed instructions. Rather, they are enacted through the routines, interactions, and material arrangements of actors working across multiple system levels. While this study engages with policy implementation in a broad sense, it adopts a policy enactment perspective to examine how policy meaning is built and reshaped in practice. In contrast to approaches that emphasize the execution of relatively stable policy goals, a policy enactment perspective foregrounds local interpretation, resource constraints, and the relational processes through which policy is translated into situated action (Bell and Stevenson, 2015; Hay, 2025). This perspective draws on scholarship that understands enactment as a context-dependent rather than linear process (Braun et al., 2010; Ball et al., 2011; Clarke et al., 2015), and it aligns with related work on policy translation and performativity across organizational settings (Elg et al., 2025; Mason and Araujo, 2021).

**Figure 1:**
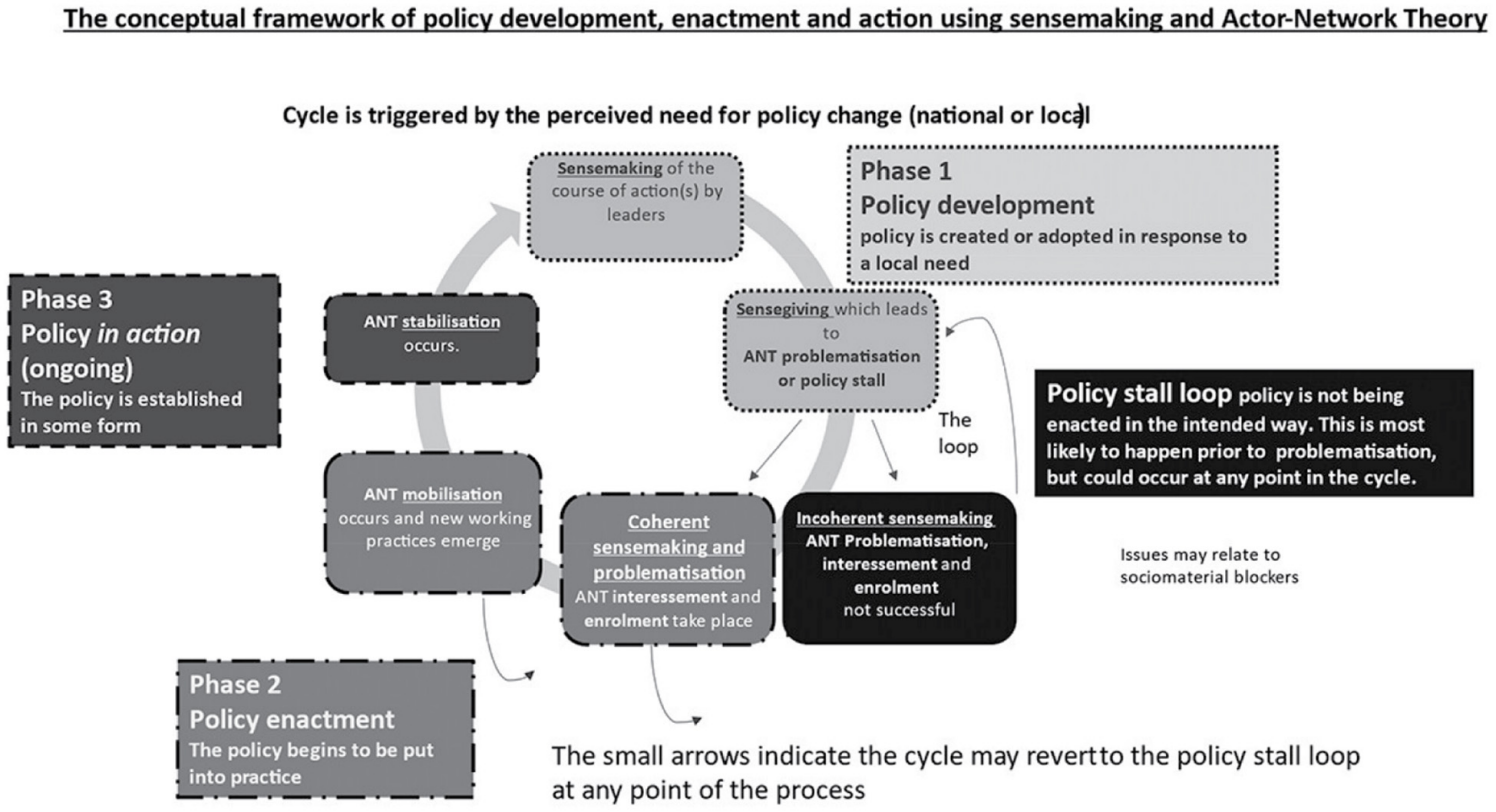
The conceptual framework of policy development, enactment and action (Hay, 2025, p. 184).

**Figure 2:**
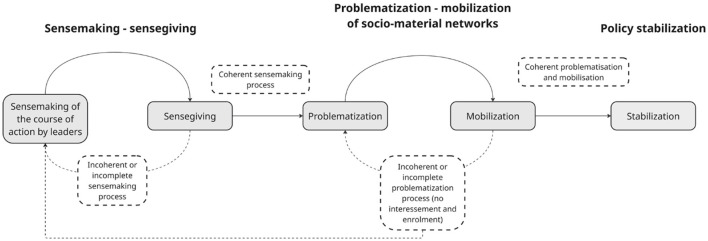
Visual reinterpretation of Hay's (2025) framework of policy development, enactment, and action.

The analytical backbone of this study is Hay's (2025) framework of policy development, enactment, and action (Figure 1), which conceptualizes enactment as a dynamic process shaped by iterative cycles of interpretation and coordination. In this paper, we re-visualized this framework (Figure 2) to examine how policy meanings and practices evolve through interconnected processes of sensemaking and socio-material reconfiguration.

### 2.2 Sensemaking and socio-material configuration

To examine how policy meanings are interpreted and aligned in practice, we draw on sensemaking theory, which explains how actors construct meaning in order to guide action (Weick et al., 2005). It is closely linked to sensegiving, defined as attempts to influence how others understand and act on an issue (Gioia and Chittipeddi, 1991). In policy contexts, sensemaking–sensegiving cycles help explain why policy intentions are taken up differently across roles and settings, particularly by middle managers and frontline staff who interpret and adapt policy in everyday work (Lipsky, 1981; Ran and Golden, 2011). Actors do not passively absorb policy meanings but actively construct what matters in their context, renegotiating values, identities, and relationships through interaction (Weick et al., 2005).

Enactment is not only shaped through actors' interpretation, but also by how practices are organized through tools, routines, and infrastructures. We also draw on a socio-material perspective informed by Actor–Network Theory (Law, 2008) to examine how documentation systems, tools, guidelines, and routines help organize practice by enabling, constraining, and stabilizing particular ways of working (Law, 1986, 2008; Cressman, 2009; Unsworth, 2024).

Hay's (2025) framework captures this through cycles of problematization and mobilization, in which actors define problems, align roles, and coordinate resources. Together, sensemaking and socio-material configuration provide a way to examine how both meaning and material arrangements shape the translation of policy into practice.

### 2.3 Systems-aware, participatory design and its role in supporting sensemaking and enactment cycles

Policy enactment helps explain how policy is interpreted and adapted but offers less guidance on how actors can work collectively across interpretive gaps. We therefore introduce a systems-aware participatory design perspective as a practical complement. Participatory and systemic design emphasize dialogue, collective sensemaking, and power redistribution in complex sociotechnical systems (Bjorgvinsson et al., 2010; Donetto et al., 2015; Blomkamp, 2022; Loi et al., 2025).

Prior literature has explicitly linked design to meaning-making, collective interpretation and decision-making, and the exploration of alternative futures (Krippendorff, 1989; Eneberg, 2012; Jones, 2018; Utterberg Modén et al., 2024). Methods such as prototyping, storytelling, visualization, and scenario work can help surface tacit knowledge, negotiate meanings, and explore alternative courses of action (Brandt et al., 2012; Eneberg, 2012; Forlano and Mathew, 2014). Participatory and systemic design have also been discussed as relevant to policymaking and social innovation, although evidence of implementation impact remains limited (Bason, 2014; Ryan and Leung, 2014; Kimbell and Bailey, 2017; Van Der Bijl-Brouwer, 2017; Van der Bijl-Brouwer and Malcolm, 2020; Blomkamp, 2022).

**Figure 3:**
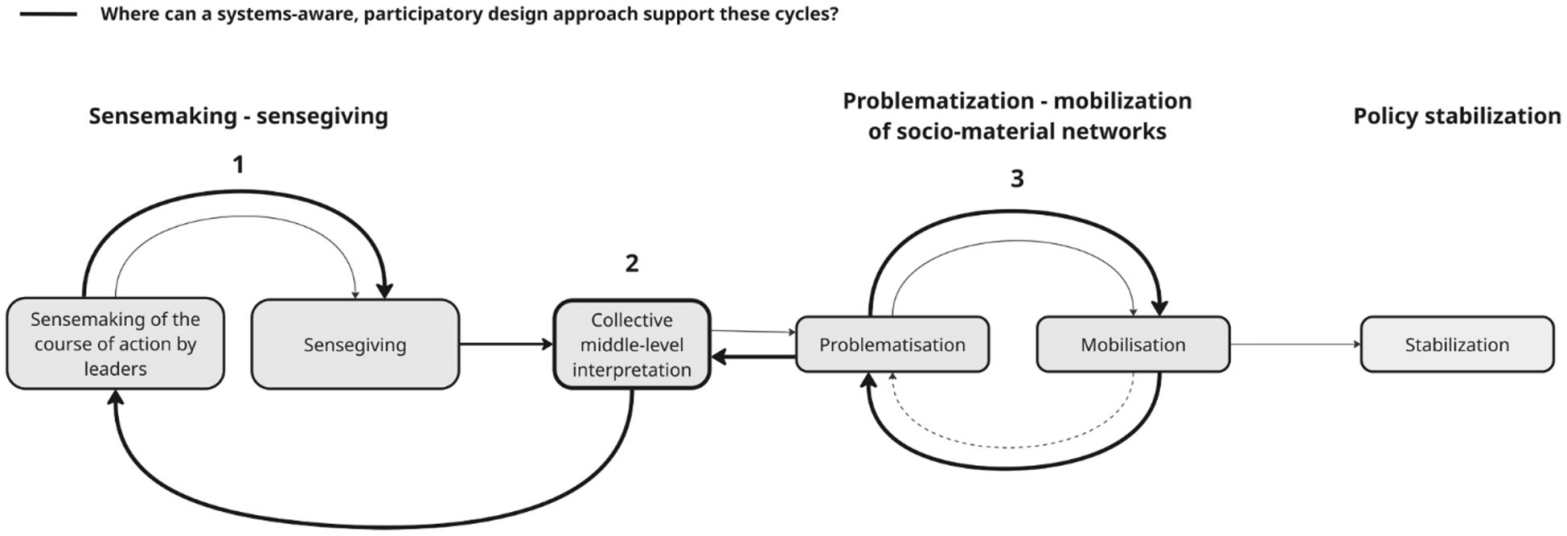
Moments where PD and SD can support the cycles of sensemaking-sensegiving and problematization -mobilization of socio-material networks.

In this study, we suggest systems-aware, participatory design as a practical complement to policy enactment to support collective sensemaking around policy (see Figure 3, number 1), help actors develop provisional shared interpretations to connect policy and action (Figure 3, number 2), and surface tensions between policy ideals and everyday work (Figure 3, number 3).

## 3 Materials and methods

### 3.1 Study context: Sweden and the implementation of PCC

Person-centered care (PCC) is a longstanding principle in Swedish health and social care policy and underpins reforms such as Nära Vård [Good and Close Care] (Swedish Government, 2018), the National Dementia Strategy 2025–2028 (Socialdepartementet, 2025), and Sörmland's Dementia Strategy 2019 (Samverkansrådet för vård och omsorg vid demenssjukdom, 2019). Although these policies differ in scope and governance level, they share a common person-centered value base and are intended to be enacted in everyday care practice. Responsibility for implementation, however, largely rests with municipalities.

However, research on Swedish health and social care suggests that policy values do not translate linearly into practice, but are interpreted and reshaped as they move across organizational levels in relation to local conditions, professional norms, available resources, and institutional logics (Czarniawska and Sevón, 2005; Elg et al., 2025). Most research on PCC implementation in Sweden has been conducted in residential care and shows that leadership practices play a central role in shaping staff wellbeing, care quality, and the sustainability of person-centered approaches (Edvardsson et al., 2014; Backman et al., 2022, 2023; Kirvalidze et al., 2024).

These challenges intersect with broader workforce pressures, including staff shortages, turnover, burnout, and declining educational levels in aged care internationally (Morris et al., 2023; OECD, 2020). Existing research also suggests that dementia-specific competence alone is insufficient if organizational conditions do not allow staff to exercise judgment, adapt care, and participate meaningfully in shaping practice (Narayanasamy and Penney, 2014).

Although a growing body of international research highlights the importance of PCC in home-based dementia care (Fazio et al., 2018; Polacsek et al., 2020; Goh et al., 2022; Marulappa et al., 2022; Xu et al., 2024), evidence remains limited on how PCC-oriented policies are enacted within municipal homecare systems, where multiple person-centered policy ambitions converge under conditions of time pressure, limited continuity, and organizational fragmentation. This makes Swedish municipal homecare a relevant setting for examining how person-centered and dementia-related policies are interpreted and translated in practice.

### 3.2 Study design

This qualitative study combined semi-structured interviews with systems-aware, participatory design workshops (Blomkamp, 2018; O'Cathain, 2025). This design was chosen to both examine how policy intentions were interpreted and enacted in dementia homecare practice and to experimentally explore how participatory methods could support collective sensemaking and policy translation.

Semi-structured interviews were used to map perceived gaps between policy intentions, organizational routines, and everyday care practices, and to examine conditions shaping enactment. Building on these insights, the workshops were designed as structured spaces for cross-level dialogue and collective reflection. Sessions combined guided reflection, small-group dialogue, scenario-based exploration, and collaborative prototyping. In this way, the study design supported both the analysis of policy–practice dynamics and the exploration of participatory processes that might help bridge abstract policy goals and situated care practice.

### 3.3 Study sample and participant recruitment

This qualitative case study was conducted in the county of Sörmland, a mid-sized region in central Sweden comprising nine municipalities with varying population sizes and service configurations. Semi-structured interviews were conducted across three municipalities within the region, while the participatory workshops were conducted in a single municipality. These municipalities differ in size and organizational capacity, allowing the study to examine policy enactment under varying local conditions within a shared regional and national policy context.

A purposive sampling strategy was used to capture perspectives across different levels of the homecare ecosystem. Participants included municipal-level managers, healthcare managers, homecare unit managers, and frontline staff from both homecare and healthcare services, and members of local dementia expert teams. While the study sought perspectives across multiple system levels, most participants were situated at the local level, where policy is translated into everyday care practice. Participant characteristics and inclusion criteria are summarized in Table 1. We collected background information on participants' professional role, sex, and years in role, with more complete information available for interview participants than for workshop participants. Among interviewees, time in role ranged from 1 to 16 years. Frontline staff had between 3 and 9 years of experience in their current role.

**Table 1:** Participant inclusion criteria.

Profile	Inclusion criteria	No (%) of participants in interviews	Sex	No (%) of participants in workshops	Sex
Policymakers	Policymakers at a regional or national level with a minimum of 3 years of experience in the development, implementation, and monitoring of policy for dementia care, aging/care for older adults, long-term care.	3 (17.6%)	Male-−1 (6%) Female-−2 (12%)	0	—
Directive/managerial roles in the social care department	Municipality level managers whose areas provide services for people with dementia living at home (e.g., social worker management, dementia management, coordinating nurses at primary care units, home care management, etcetera) Dementia expert teams are included in this category	5 (29.4%)	Female-−5 (29%)	10 (23.8%)	Male-−2 (6%) Female-−8 (25%)
Homecare unit managers and coordinators	Professionals who have at least 2 years of experience organizing/managing homecare provision for people with dementia	5 (29.4%)	Female-−5 (29%)	11 (26.2%)	Male-−1 (1%) Female-−7 (22%)
Frontline homecare staff	People who have at least 1 year of experience providing care services for people with dementia living at home (assistant nurses, registered nurses, occupational therapists, rehabilitation therapists, etcetera)	4 (23.53%)	Male-−2 (12%) Female-−2 (12%)	21 (50%)	Male-−3 (9.5%) Female-−11 (34.5%)

Recruitment was facilitated through municipality managers and members of local dementia expert teams, who distributed the study invitation across homecare units. Interested participants were subsequently contacted by the first or second author. While recruitment strategies were similar across data collection phases, the interview and workshop samples were not identical. A small number of participants took part in both interviews and workshops; however, the workshops involved a broader group of participants from similar professional roles and organizational levels within the same municipal context. Written informed consent was obtained from all participants and returned directly to the first author, either in paper form or electronically.

### 3.4 Data collection

Three complementary sources of data were collected: semi-structured interviews, participatory design workshops, and Patient and Public Involvement (PPI) activities with people living with dementia and unpaid carers.

#### 3.4.1 Semi-structured interviews (n = 17)

Semi-structured interviews were conducted by trained interviewers and lasted between 50 and 75 min. All interviews were audio-recorded with participant consent and transcribed verbatim. An interview guide (see Supplementary materials) was used to ensure consistency while allowing flexibility to explore participants' experiences of policy translation, organizational constraints, and care practices in depth. The interview guide was initially developed by the first author based on the study's theoretical framework and existing literature on the implementation of person-centered dementia care (Brooker, 2007; Hedman et al., 2022; Lee et al., 2023). The themes and questions were subsequently reviewed and refined with PPI groups, including people living with dementia and unpaid carers, to ensure relevance, clarity, and sensitivity to lived experience. Interviews were conducted online or in person, and in English, Spanish, or Swedish, according to participant preference.

#### 3.4.2 Participatory design workshops (n = 14–18 per workshop)

Two participatory design workshops were conducted to foster cross-level dialogue on how local policy goals are enacted in everyday homecare practice. Each workshop lasted approximately 150 min. The workshops were designed as complementary sensemaking spaces with different analytical entry points.

Workshop 1 followed a top-down approach: it began with selected policy extracts and invited reflection on policy intent, expectations, and interpretive tensions. Workshop 2 followed a bottom-up approach: it began from an existing routine and tool, the Life Story, and worked back to the policy intentions underpinning its use. The Life Story (LS) is a standardized, paper-based document intended to support person-centered care by capturing information about care recipients' life histories, preferences, routines, and relationships (McKeown et al., 2010; Möllergren and Harnett, 2024).

Workshop activities and materials were developed iteratively, drawing on participatory design literature (Brandt et al., 2012; Matos-Castaño et al., 2020; Utterberg Modén et al., 2024), and were refined in collaboration with local dementia expert teams and unpaid carers to ensure contextual relevance. Both workshops were facilitated by the first author (AN), with support from local co-facilitators during small-group activities. While overall framing and instructions were provided in English, small-group discussions, materials, and presentations were conducted in Swedish to reduce participation barriers and support nuanced discussion of local practices. Workshop data included written artifacts (e.g., post-its, canvases, prototypes), photographs of group outputs, facilitator field notes, and audio recordings of whole-group discussions where consent was provided. The workshop protocol can be found in Supplementary materials.

#### 3.4.3 Patient and public involvement

Six sessions were held with people living with dementia and unpaid carers: two with the European Working Group of People with Dementia, one with the European Working Group of Carers of People with Dementia, and three sessions with a local group of unpaid carers in Sweden. These activities informed the development of research materials and were also used to discuss and validate emerging findings during the analytic process.

### 3.5 Data analysis

All transcripts were translated into English or Spanish, de-identified, and managed using NVivo 15 (Lumivero, 2025). Analysis proceeded in four stages across interview and workshop datasets. For the interview material reported here, an initial inductive stage guided the generation and refinement of codes and the grouping of related codes into clusters. A subsequent abductive stage re-examined the clusters and coded data through a policy enactment lens to develop the final themes. Codes that did not address the research questions or align with the policy enactment framework were excluded. Workshop data was then analyzed separately using the framework method, before findings from both datasets were compared and integrated. Coding, clustering, and theme development were led by the first author and discussed regularly with co-authors to refine interpretations and consider alternative explanations.

#### 3.5.1 Stage 1: inductive coding and clustering of interviews

Interview data was first examined inductively to develop an exploratory understanding of participants' experiences of person-centered care, dementia-related values, everyday care practices, routines, and policy translation. Meaningful segments were coded, codes were refined through constant comparison within and across interviews, and related codes were grouped into provisional clusters.

To strengthen the analysis, a second co-author (DS) reviewed 10% of the transcripts and discussed coding choices and emerging interpretations with AN. These discussions, together with wider analytic meetings, functioned as peer debriefing rather than inter-coder reliability testing (Braun and Clarke, 2022). While this stage produced a descriptive and practice-oriented analytic structure, it did not fully explain how policy intentions were translated across system levels. This analytic gap motivated the abductive phase.

#### 3.5.2 Stage 2: abductive analysis through a policy enactment lens

To move beyond description, we undertook a theory-informed abductive analysis (Tavory and Timmermans, 2014; Thompson, 2022). Clusters, coded extracts, and full transcripts were revisited through Hay's (2025) policy enactment framework as a sensitizing lens to identify tensions, ambiguities, and patterns not fully explained by the initial descriptive analysis (Vila-Henninger et al., 2024). This stage focused on identifying tensions and patterns related to sensemaking–sensegiving and socio-material configuration across system levels. Clusters that spoke to related enactment processes were reorganized into more interpretive sub-themes and themes, leading to the six conditions reported in this paper. Table 2 illustrates this progression for one worked example, showing how inductively developed codes were grouped into clusters and subsequently re-specified abductively as a sub-theme and overarching theme within the policy enactment cycles. Table 3 provides examples of inductive codes and their corresponding text segments. Preliminary themes were discussed within the research team and presented to a local unpaid carer group as part of PPI-based validation.

**Table 2:** Example of the analytic process.

Theme (developed abductively)	Sub-theme (developed abductively)	Clusters (developed inductively)		Codes (developed inductively)
Conditions influencing the problematization—mobilization cycle	Resource-constrained and undervalued conditions of homecare work	Financial challenges for homecare municipal and unit managers		Large costs for prof carers education
				Flexibility entails increasing costs
				Difficulties attracting new workforce to the geriatric area
				Lack of evidence on the need to increase homecare funding
		Challenges related to workforce stability within the homecare unit	Organization of workforce	Challenge to organize the frontline staff while not having a fixed number of user hours
				Assistant nurses' rotation
				Need to limit the coverage of the service to secure continuity
			Collaboration and shared mindset	Challenges when there are many different carers, which means different mindsets
				Unit manager rotation hinders continuity
		Impact of challenges in coordination with other services		Impact in the implementation of the coordinated individual plan
				Need for a good understanding and delimitation of everyone's role
		Time constraints		Limited time = limited conversations
				Importance of having more time to work with people with dementia
		Negative perceptions of the homecare service and workers		Generalized pessimism from the care receivers when entering the homecare system
				Migrant workers perceived as unable to take care of local people
		Low symbolic and economic recognition of the value of homecare		Low salaries compared to other personnel in social care services
				Being perceived as a second-tier option

**Table 3:** Example of inductive codes with corresponding text segments.

Codes	Text segment from data
Large costs for prof carers education	*The big problem for us, as I see it, is the money situation, the economy. We have to keep the unit financially stable, and at the same time we are expected to give staff education and provide them with the right tools through training. Sometimes we are told, “Okay, we will give you some extra money if you send your staff to this education session,” but the problem is that the same work still has to be done on the unit within the same eight-hour day. That pressure is always there*. (Participant 3, Unit manager)
Lack of evidence on the need to increase homecare funding	*They need they want us to show that the time of home care is increasing to get more fundings, but we can't really show that. Funding must come first, so that we can increase the hours and then show why they are necessary*. (Participant 8, Municipal manager)
Need to limit the coverage of the service to secure continuity	*The X group [specialized in dementia] is quite small and has a limited number of care recipients. That is the key: being able to keep control of the number of care recipients. If it grows too much, you lose continuity. We had a period when the pressure was high and we almost lost it. We had almost 60 care recipients. It was too much. There were not enough staff in the X group, so we had to bring in volunteers to meet the needs. And it is not the same with volunteers… they do not have the same experience*. (Participant 4, Assistant nurse)
Unit manager rotation hinders continuity	*I think one of the main issues is that people don't stay at work for very long. For example, a lot of bosses [unit managers]. Yeah, they switch places they switch the course [of action] completely… For example, we have a boss who was a policeman and started work as a boss. So, it takes time for them to get the knowledge [they need]. And during that time, things can happen, and things can go wrong. Even if we have guidelines here, it's a lot of things to catch up with*. (Participant 2, Dementia specialist)
Limited time = limited conversations	“*And I feel like we don't really have the time to talk to them enough. At least sometimes, we should be able to sit there and talk to them for a bit before going to the next one, you know? […] It is about completing our tasks, but it also should be about building a relationship and help them to have a good start of their day”* (Participant 4, Assistant nurse)

#### 3.5.3 Stage 3: framework analysis of participatory workshop data

Data generated through the participatory design workshops were analyzed separately using the framework method (Ritchie and Lewis, 2003; Gale et al., 2013). This approach enabled systematic comparison across workshop groups and activities while retaining close connection to participants' discussions. Whole-group presentations of outputs were audio recorded, transcribed, and translated into English, as well as relevant written materials. Translations were checked by one of the local Swedish facilitators, and debrief sessions were held after each workshop to consolidate facilitator notes and initial impressions.

Analysis was mainly conducted by AN and included familiarization with workshop materials, development of an analytic framework, indexing of data, and charting into a matrix structured around workshop phases and group outputs. The framework was refined iteratively, combining inductive identification of issues with deductive interpretation informed by the policy enactment perspective. A final analysis session with co-facilitators was held to discuss and validate the main findings and conclusions.

#### 3.5.4 Stage 4: cross-dataset integration

Finally, insights from the interview and workshop analyses were compared and integrated. This analysis examined convergence, divergence, and complementarity across the two phases, and supported identification of shared, provisional interpretations connecting policy ambitions with situated care practice. Findings were discussed with the local co-facilitators and the authors for triangulation and refinement.

#### 3.5.5 Reflexivity and language considerations

Because data were generated in Swedish, Spanish, and English, translation was an important methodological consideration. Interviews and workshop materials conducted in Swedish were translated into English for analysis, which may have entailed some loss of nuance. To mitigate this risk, translations were checked during data collection, and interpretation was discussed across the research team and with local collaborators. During the workshops, facilitator-checked translations and post-workshop debriefs were used to strengthen our interpretation.

The research team's positionality also shaped this study. The team combined complementary perspectives, including non-Swedish service design researchers and Swedish researchers with backgrounds in care sciences, enabling interpretations to be both challenged and grounded in local practice. The first author (AN), as a non-Swedish and migrant researcher with a background in psychology and service design, occupied an outsider–facilitator position that supported questioning taken-for-granted practices and appeared to facilitate openness among some participants with migrant backgrounds. In contrast, interviews conducted by the Swedish co-author (JL) brought an insider perspective, supporting contextual understanding and rapport while also carrying the risk of reinforcing implicit assumptions.

In the participatory design workshops, AN was an active facilitator, influencing how discussions unfolded. Analytic interpretations were shaped through ongoing dialogue within the research team and with local collaborators and were addressed through discussion and peer debriefing, acknowledging that findings are co-constructed and situated.

## 4 Findings

**Figure 4:**
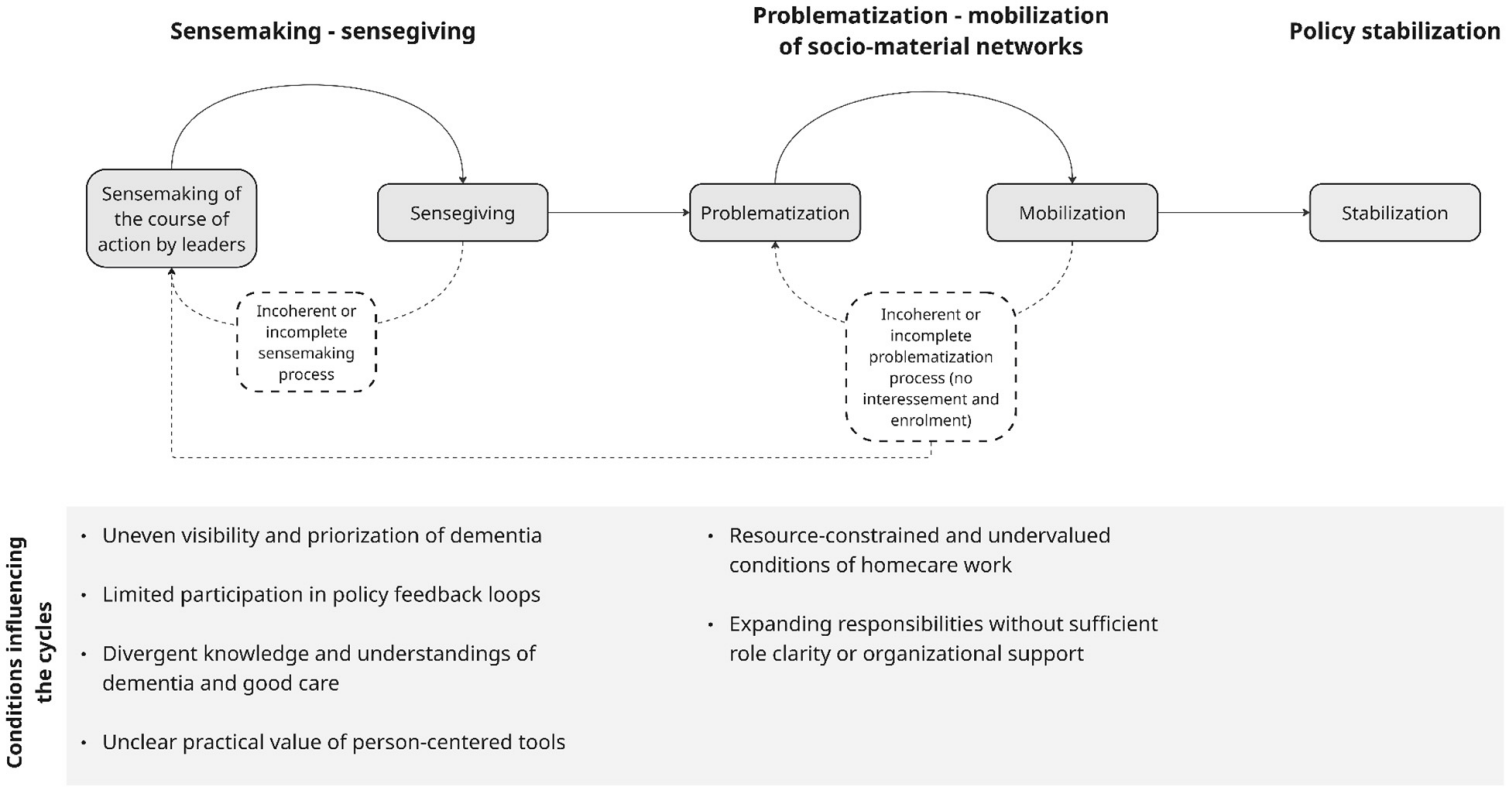
Conditions influencing sensemaking-sensegiving and network problematization-mobilization cycles.

The first part of the results presents findings from the semi-structured interviews, illustrating six conditions influencing policy enactment in dementia homecare. Figure 4 summarizes these conditions. The second part focuses on findings from the participatory design workshops, which explored how actors collectively interpreted policy goals and routines and developed more practice-oriented responses.

### 4.1 Conditions influencing the sensemaking–sensegiving cycle

As shown in the left section of Figure 4, participants described several conditions that shaped how person-centered and dementia-related policy goals were understood, communicated, and related to everyday homecare practice. Through interviews, these accounts pointed to four patterns: uneven visibility and prioritization of dementia policy, limited participation in policy feedback loops, differences in knowledge and understandings of dementia and good care, and uncertainty about the practical value of person-centered tools.

#### 4.1.1 Uneven visibility and prioritization of dementia

Although broad person-centered ambitions, particularly through Nära Vård, were widely recognized across municipalities, dementia-specific policy goals were less visible in local agendas and everyday homecare practice. Most participants across managerial and frontline roles could articulate general principles of person-centered care, yet dementia-specific policy goals were weakly articulated or not explicitly prioritized at the municipal level. Some municipal managers acknowledged that dementia did not have a clear local focus, despite an increasing number of people receiving homecare with cognitive impairment.

This limited visibility was also reflected in participants' familiarity with policy documents. Aside from members of local dementia expert teams, most interviewees reported limited or no awareness of national or regional dementia strategies. As one participant explained, dementia-related initiatives introduced at the county level were not consistently taken up locally:

“*Not everything that has been suggested at county level on the topic of dementia care is applied locally. There is resistance… sometimes because of money, sometimes because of time. The final consequence is that our local strategy has not been reviewed in years.”* (Participant 2, Dementia specialist, 11 years in their role)

Participants also described variation in how policy ambitions were translated into everyday guidance. New routines and person-centered principles were mainly communicated through unit managers, quality assurance coordinators, and sometimes local dementia expert teams, but the frequency and depth of such discussions differed across units. In this context, several participants noted that leadership support often took the form of broad goals (such as person-centeredness, integrated care or continuity) without clarifying how these should be enacted in practice. One unit manager described this as a gap between clearly stated ambitions and limited support for implementation:

“*The goal is that by 2027 we will have developed early, coordinated, health-oriented support […] It all sounds very good. […] But how I do this, no one tells me. I am expected to create the activities myself. And that ‘how' does not look the same everywhere.”* (Participant 11, Unit Manager, 4 years in their role)

Taken together, participants described dementia-specific policy goals as inconsistently prioritized and insufficiently translated into everyday guidance.

#### 4.1.2 Limited participation in policy feedback loops

Participants across roles described policy translation as largely top-down, moving from national to regional to local levels with few mechanisms for bottom-up input or collective reflection. Managers noted that while local dementia expert teams could sometimes provide written comments on strategies, their involvement rarely extended to ongoing discussions or shared interpretation.

At unit level, staff participation in reviewing or revising routines was described as inconsistent and often dependent on the practices of individual managers rather than embedded in regular structures. As one unit manager explained, staff perspectives were recognized as important because of their close connection to practice, yet were not routinely included:

“*When a routine is up for review, it is usually me who is expected to read it and give feedback. Sometimes I choose to share it with an employee and ask what feels good or what is missing. But I don't think everyone does that […] employees have a real connection to what is happening in practice, so their perspective is very important. But we don't always get it. We rarely do.”* (Participant 10, Unit Manager, 3 years in their role)

Participants across levels described limited involvement of people with dementia and their relatives in shaping care practices beyond initial assessment meetings. Barriers such as late introduction of services, limited awareness of rights, and low system flexibility were reported as factors restricting participation. These experiences pointed to a gap between those involved in defining policy goals and those involved in delivering and experiencing care, with consequences for how policy was understood and taken up in practice.

#### 4.1.3 Divergent knowledge and understandings of dementia and good care

Participants across managerial and frontline roles described differences not only in dementia-related knowledge, but also in how dementia, participation, and good care were understood in practice. Several municipal and unit managers acknowledged having limited dementia knowledge, while also pointing to perceived skill gaps among care staff. However, participants did not describe competence needs in the same way. Some municipality managers emphasized language barriers and insufficient knowledge, whereas unit managers more often highlighted communication, relational work, and the ability to assess changing needs over time. One unit manager described these relational skills as central to person-centered dementia care:

“*If you don't have education in how to talk to a care recipient, you don't know how to ask questions. Before, the main thing in the work was to help with the shower or other practical things. Now we expect staff to sit down, ask questions, and build a relationship. But we don't give them the training they need.”* (Participant 3, Unit Manager, 4 years in their role)

Participants also described differing assumptions about dementia, aging, and “good care” that shaped how person-centered principles were interpreted. Dementia was often framed in medical terms, and participation was commonly understood as something occurring outside the home through structured activities or meetings rather than within everyday care interactions. As one municipal manager explained:

“*[…] in Sweden, we still look at older people as a homogeneous group. […] and dementia is still very much seen as an illness… we don't talk about it in terms of citizenship or participation. We're still in the illness, in the medical perspective.”* (Participant 8, Municipal manager, 1 year in their role)

Frontline staff further reported differing views on what good care meant, with some situations described as favoring doing tasks for the person rather than supporting independence. These expectations were sometimes reinforced by relatives.

#### 4.1.4 Unclear practical value of person-centered tools

Participants described variation in how person-centered tools were perceived and used in everyday practice. This was particularly evident in discussions of the Life Story tool. While some participants viewed it as a resource for understanding care recipients' preferences and histories, others questioned its relevance in the daily care of people with dementia. For some frontline staff, usefulness was judged mainly in relation to whether a tool supported immediate decision-making in the situation at hand. In that context, some staff felt that the Life Story directed attention to the person's past rather than helping them respond to present needs. One assistant nurse explained:

“*They told us that we had to ask the care recipient if they wanted to write their life story. Many people with dementia get help from their relatives. And supposedly that helps staff understand who the person was, but at the same time… it actually makes you lose focus on who they are today […] I see the patient as they are today. I don't read the Life Story because I adapt to who the patient is at that moment. I've never really focused on what they were before.”* (Participant 6, Assistant nurse, 8 years in their role)

Participants also described uncertainty about how the purpose of the tool had been communicated across teams. In some cases, it was unclear how the information should be gathered, updated, or translated into care. As a result, the same tool could be regarded as meaningful in one unit and marginal or impractical in another.

### 4.2 Conditions influencing the problematization and mobilization of networks

Participants also described a set of conditions that shaped how person-centered dementia care could be organized and carried out in practice. Across interviews, these accounts pointed to two connected patterns: first, homecare was often delivered under resource-constrained and socially undervalued conditions; second, policy ambitions were associated with expanding responsibilities and new forms of collaboration that were not always matched by clear role definitions or organizational support.

#### 4.2.1 Resource-constrained and undervalued conditions of homecare work

Across roles, participants consistently described time pressure, workforce instability, and limited continuity as central conditions shaping everyday homecare. High staff turnover, reliance on temporary workers, and short, fragmented visits were reported as ongoing challenges that affected both coordination among staff and opportunities to build trust with people living with dementia and their families. Frontline staff often described not having enough time to combine practical tasks with relationship-building. As one assistant nurse explained:

“*And I feel like we don't really have the time to talk to them enough. At least sometimes, we should be able to sit there and talk to them for a bit before going to the next one, you know? […] It is about completing our tasks, but it also should be about building a relationship and help them to have a good start of their day.”* (Participant 4, Assistant nurse, 9 years in their role)

Participants also noted that limited resources affected how new routines and tools were received in practice. Frequent introduction of initiatives, combined with lack of time and staffing, was described as contributing to skepticism toward new practices and reducing opportunities to reflect on or adapt them to individual needs. Some municipalities were described as exploring more flexible approaches, such as reducing strict time allocation, but these were presented as exceptions rather than common practice.

Alongside these organizational pressures, participants described homecare as a form of work that was often undervalued within the broader care system and in public discourse. Some participants perceived that homecare had a lower status than other forms of care, despite the increasing complexity of supporting people living with dementia at home. Frontline staff also described negative public perceptions of homecare services, which shaped expectations and interactions with care recipients and families. One assistant nurse described this in the following way:

“*What hemtjänsten [home care workers] have is that […] it's like people have been told that we do whatever we want in people's homes… that we're the worst group you could want to bring into your home. If a car drives by fast, it's like ‘oh, those are the hemtjänsten'; if they arrive at a different time than expected, ‘yes, that's the hemtjänsten.' And that reputation is similar everywhere […] Many older people wait as long as possible before accepting help, and when they do, it's almost at the very last stages.”* (Participant 6, Assistant nurse, 8 years in their role)

Unit managers and staff further described how delayed uptake of services often meant that support was introduced when needs had already become more complex, affecting both the intensity of care required and the possibilities for building relationships over time. Some participants also emphasized that low recognition of homecare work was intertwined with migration and ethnicity. Staff with migrant backgrounds reported experiences of discrimination and low recognition, and some participants suggested that homecare workers were often perceived as a homogeneous group of migrants, reinforcing stereotypes about competence, cultural sensitivity, and professionalism.

#### 4.2.2 Expanding responsibilities without sufficient role clarity or organizational support

Participants across all levels described ongoing changes in what homecare work required under policies promoting PCC. These changes included greater expectations around documentation, communication with families, relational work, early support, and collaboration across professional groups. However, participants also reported that these expanding responsibilities were not always matched by clear role definitions, agreed responsibilities, or established ways of working together.

Several participants described tensions between administrative requirements and the relational aspects of care, particularly in relation to documentation and standardized routines. While documentation systems were recognized as important for ensuring accountability, transparency, and continuity, some managers noted that increasing documentation demands shaped daily work in ways that prioritized task completion. One unit manager explained how these changes had altered expectations of frontline staff:

“*… you have to document something almost every day: how the care users are, how they feel, what we did in their homes, and whether we see any difference in their situation. […] There is a lot of documentation. And now, from the government [National board of health and welfare] they are not only expecting us [homecare services] to give care, they are also expecting us to work in a new way. The role of assistant nurses has changed a lot. It's not only giving medicine or helping with toilet needs anymore. You must write, you must talk more with families, and you must ask about how the person lived before, what they liked, who they were… It is a lot more.”* (Participant 11, Unit Manager, 4 years in their role)

Unit managers and frontline staff described how documentation and routines structured care work, but also how activities related to relational care, such as building trust, adapting to changing needs, or engaging in meaningful conversations, were less visible and more difficult to prioritize within existing schedules.

Participants also described difficulties in coordination across professional groups and municipal services. Expectations for closer collaboration, more proactive support, and greater continuity across the care trajectory were reported, but responsibilities, mandates, and decision-making authority were not always clear. Some frontline staff and unit managers described difficulties in transferring knowledge across roles, especially when multiple professionals were involved over time but had limited opportunities to interact. Collaboration across services was also described as initially producing concern about professional boundaries and accountability. One dementia specialist gave the following example:

“*When collaboration across services was introduced, there was resistance from case managers… They were afraid that someone coming along would not understand their profession and might unintentionally promise things to families […] Working together led to many ‘aha' experiences […] It was only through following case managers in practice that we understood the complexity of their role… Over time, and by being careful not to cross professional boundaries, collaboration gradually improved.”* (Participant 12, Dementia specialist, 16 years in their role)

These findings highlight how changes in policy expectations were accompanied by evolving roles and forms of collaboration, which were not always clearly defined or supported in practice.

### 4.3 Participatory design workshops: fostering sensemaking and bottom-up policy translation

Building on the interview findings, two workshops were designed to examine how actors across organizational levels interpreted policy goals in relation to dementia homecare and how participatory methods could support that process. Workshop 1 began from a policy concept and worked toward local interpretation, while Workshop 2 began from an existing routine, the Life Story, and worked back toward the policy intentions associated with its use. Drawing on systems-aware participatory design methods, we created opportunities for actors to jointly reflect on policy meanings, constraints, and possibilities and to experiment with alternative interpretations grounded in everyday practice. A central aim was to support the articulation of middle-level interpretations: shared, provisional understandings that translate abstract policy goals into locally meaningful orientations for action in dementia homecare practice.

#### 4.3.1 Workshop 1: from policy intent to local interpretation and practices

Workshop 1 focused on the policy concept of co-creation in dementia homecare. Participants used a policy extract and translated this concept into concrete practices, engaging in the sensemaking–sensegiving cycle. Across groups, the interpretation and operationalization of co-creation varied (see Table 4).

**Table 4:** Synthesis of outputs from workshop 1, per team.

Data categories	Team 1	Team 2	Team 3
Main topics of discussion	Main focus: knowledge, clarity, and respectful interaction Key gaps identified: • Fragmented and unclear information pathways for people with dementia • Risk of infantilization and loss of adult identity • Limited attention to non-verbal communication and emotional cues	Main focus: time, proactivity, and structural constraints Key gaps identified: • Insufficient time and flexibility to support co-creation • Reactive rather than proactive care trajectories • Tensions between person-centered care, legal frameworks, and organizational routines • Relationship with relatives recognized as very relevant for decreasing anxiety and encouraging co-creation, but highlighted its complexity	Main focus: relational work with relatives and adapting to change Key gaps identified: • Misalignment between staff, relatives, and the person with dementia • Assumptions about needs rather than listening to lived experience • Limited adaptation to changing priorities over time
Middle-level interpretation	Supporting people with dementia to become co-creators of their own care means listening, but also to listen to what is not being said.	Supporting people with dementia to become co-creators of their own care means involving relatives and the person in the team, meeting the individuals where they are [cognitive capacity], following up and adapt plan and support, coordinating professional perspectives and increasing dementia/related knowledge through team collaboration.	N/A
Main attributes highlighted	•Involving the person by showing, explaining, and confirming how they prefer things to be done. • Sensitivity to non-verbal communication, including body language and emotional cues. • Careful and respectful introductions when new staff or new tasks are introduced into the home. • Creating participation with respect for both the individual and their home. • Providing information in different formats (visual, verbal, and written) depending on the person's needs.	•Support the early involvement of both the person with dementia and their informal carers as part of the care team. However, it would be important to highlight roles and limits of informal carers. • Emphasizing curiosity in relation to quality, competence, interest, and how the work contributes to Sense of Coherence (SOC), alongside a person-centered and rehabilitative approach. • Highlighting practical practices such as checking the fridge, monitoring baseline weight, and encouraging the person to have their own scale at home to follow nutrition and health over time.	•Use of supportive tools (e.g., digital or visual aids) to support communication and shared understanding, including interpreter functions when needed. • Practical strategies for understanding daily life, such as observing eating patterns or routines, rather than relying only on formal assessments. • Reiterating core themes of participation, respect, and meeting the person where they are.

Team 1 emphasized communicative and relational practices, and focused on knowledge, fragmented information pathways, risks of infantilization, and limited attention to non-verbal communication and emotional cues. The group articulated a middle-level interpretation of co-creation as “*listening to what is not being said,”* emphasizing sensitivity to body language and flexibility to adapt information format depending on the person's needs.

Team 2 highlighted temporal and organizational conditions shaping co-creation. Participants focused on time and coordination constraints, lack of flexibility, and tensions between ideals, legal frameworks, and organizational routines. Their middle-level interpretation framed co-creation as a coordinated, team-based process that requires meeting individuals where they are cognitively, following up over time, and aligning professional perspectives through collaboration.

Team 3 emphasized relational work with relatives and adaptation to change over time. Their discussions highlighted misalignments between staff, relatives, and the person with dementia, as well as the risk of assumptions about needs replacing active listening. This group proposed attributes related to continuous adjustment, use of supportive communication tools, and attention to everyday routines as sources of understanding.

Overall, participants described co-creation as a situated and relational practice, shaped by communication, time, coordination, and ongoing adjustment. These domains did not converge into a single interpretation but reflect the conditions each team viewed as most critical for enabling co-creation in dementia care, highlighting variation in how policy concepts are understood and negotiated across roles and settings.

#### 4.3.2 Workshop 2: from routine to policy intent

**Figure 5:**
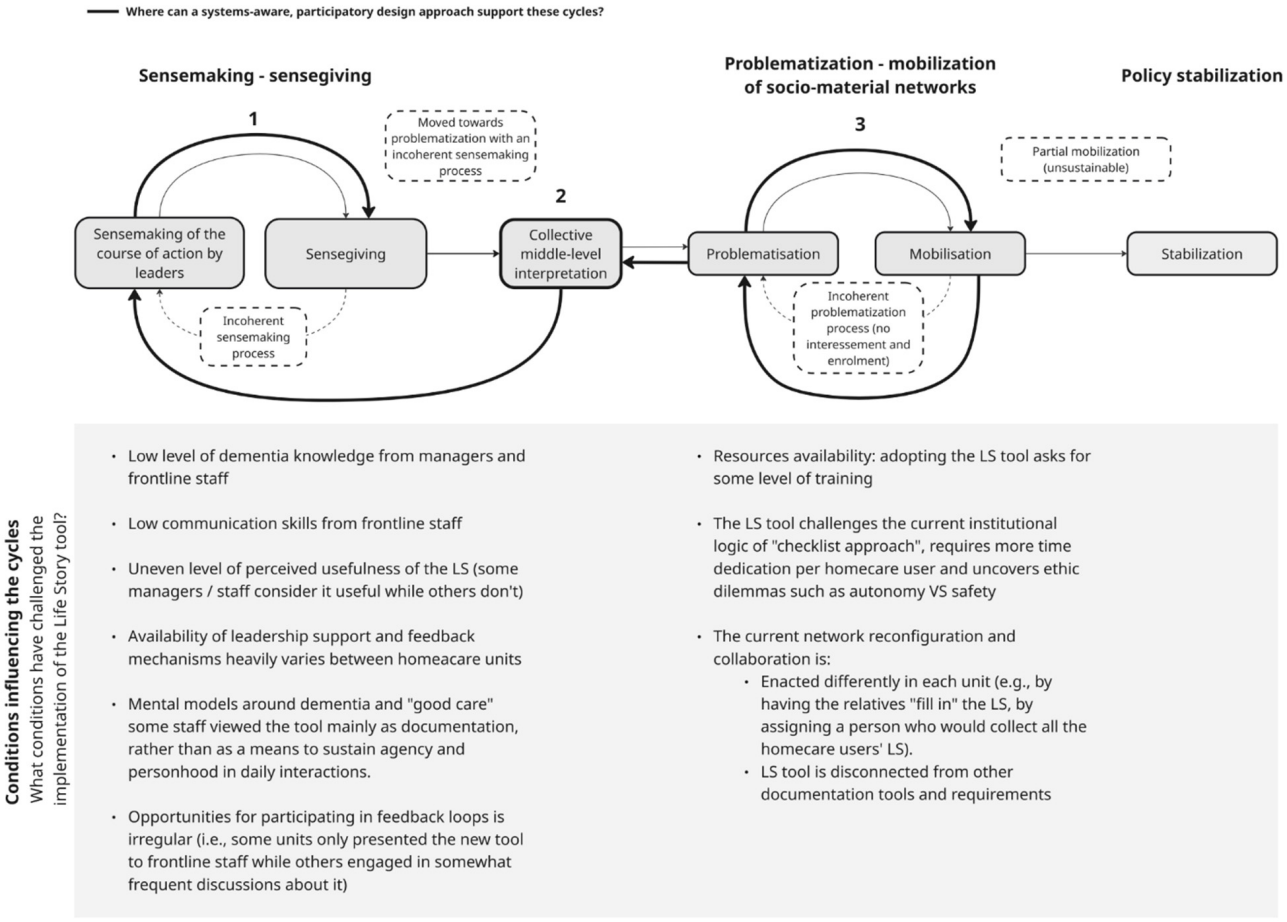
Conditions identified in the enactment of the Life Story tool and the participatory design activities facilitated during Workshop.

Workshop 2 examined how the Life Story (LS) tool is used in practice and how participants understood its role in relation to person-centered care. As shown in Figure 5, the workshop engaged with both the interpretation of the tool's purpose (sensemaking–sensegiving) and the ways in which it is adapted and used in practice (problematization and mobilization). Participants identified four main challenges: unclear understanding of the tool's purpose and its link to care outcomes; communication difficulties when eliciting meaningful information; organizational barriers such as time pressure, limited continuity, and fragmented workflows; and uncertainty about handling sensitive information.

In response, participants explored alternative ways of using or adapting the tool (Figure 5, moment 3). These included sensory and activity-based approaches to gather personal knowledge, modular and multimodal formats adapted to different stages of dementia, alternatives to standardized templates (e.g., photo-based or visual formats), and organizational adjustments such as earlier use of the tool, clearer responsibility allocation, and distributed documentation over time (see more details in Table 5).

**Table 5:** Outputs from prototyping activity in workshop 2.

Data categories	Team 1	Team 2	Team 3	Team 4
Main topics of discussion	How do we create a Life Story when verbal storytelling is not possible? How to capture meaningful personal knowledge when service users cannot speak or lack family advocates. Standard Life Story questions are ineffective for people with advanced dementia.	How can one system support life stories across all dementia stages? One single standardized Life Story format cannot serve people at different stages of dementia.	How do we make the Life Story meaningful instead of bureaucratic? • A single fixed template conflicts with PCC values. • Existing tools over-prioritize narrow life categories (e.g., military service) and miss crucial life experiences.	How do we build organizational capacity to do Life Stories properly? Life Stories fail because staff lack time, trust relationships, conversational skills, and structured processes.
Focus of the solution	•Sensory and experiential engagement (photos, humor, tactile objects, scents, music). • Trial-and-error exploration to identify preferences. • Building knowledge directly through shared activities and relationship-building. • Ensure the Life Story feeds into the care plan, not the other way around.	Adapt tools depending on: • Communication abilities (verbal vs. non-verbal). • Presence or absence of relatives. • Cognitive capacity. • Combine narrative storytelling for early stages with sensory methods for later stages. • Use recording tools (audio/video) to reduce written burden. • Treat the Life Story as a “living document” shared across staff teams.	Replace templates with: • Modular formats (themes, symbols, sensory objects). • Mind-maps, photo albums, memory boxes. • User-defined themes (family, work, loss, migration, etc.). • Emphasized meaning-making in everyday care, not documentation completion. • Symbols for quick preference recognition.	•Formal observation periods before documenting. • Split story-building over time rather than in one session. • Training staff in open questioning, observation skills, person-centered communication • Clarify responsibility: designate staff who have interest and competence. • Use the tool preventively: start at diagnosis or earlier (e.g., age 65).
Images of prototype	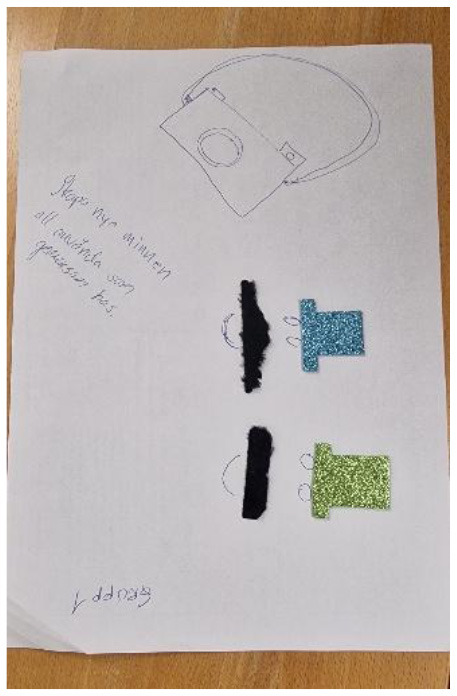	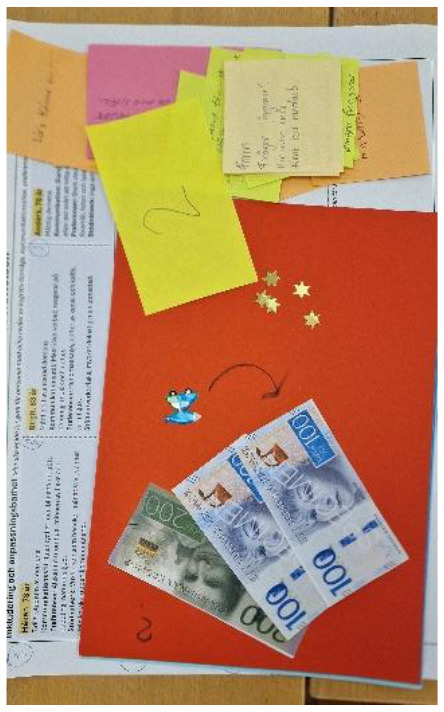 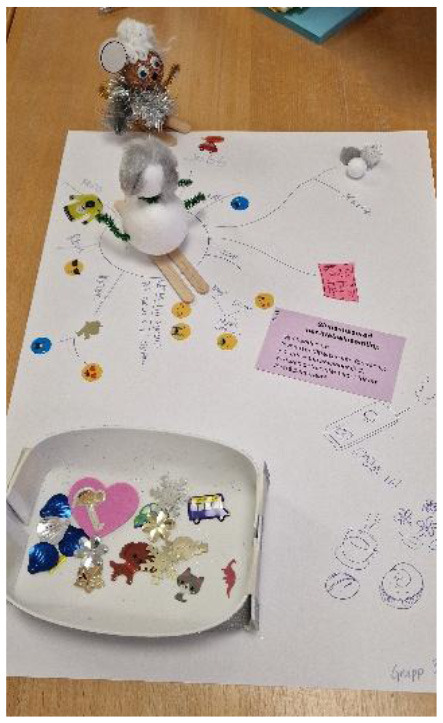	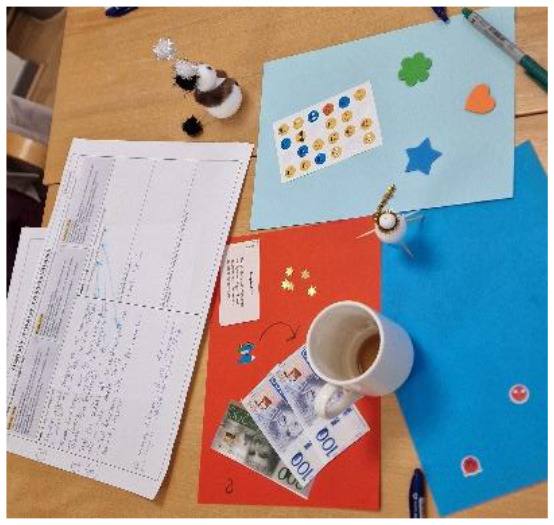	

The prototypes did not align into a single solution or shared redesign of the Life Story but represented different attempts to reframe the problem and reconfigure elements of the socio-material configuration surrounding the tool, including roles, artifacts, skills, and workflows. Participants converged around a set of key attributes that the Life Story should have and a set of surrounding conditions that should be in place, if the tool was to be more clearly connected to policy goals and more understandable and feasible for frontline staff (Figure 5, moment 2).

Taken together, the workshops supported collective reflection around policy concepts and everyday routines. Participants articulated shared concerns, compared perspectives across roles, and developed provisional interpretations that connected policy ambitions with local practice. Across both workshops, participants' discussions indicated that addressing enactment challenges would require sustained and iterative engagement beyond single-session interventions.

## 5 Discussion

This study examined how person-centered dementia policy is enacted in Swedish municipal homecare and how participatory, design-driven methods may support that process. Our findings suggest that the main challenge is not a simple lack of support for person-centered care as a value. Rather, the challenge lies in how policy ambitions are interpreted, translated, and coordinated across system levels under conditions marked by fragmented responsibilities, uneven dementia-related knowledge, limited feedback mechanisms, and resource pressures in everyday work. A policy enactment perspective was therefore useful because it directed attention not only to whether policy was implemented, but to how policy meaning was formed and re-formed in practice.

A first contribution of the study is to show that enactment depends heavily on alignment between abstract policy ambitions and the organizational conditions of homecare. Participants across roles generally expressed support for person-centered care, yet dementia-specific policy goals were not always visible, consistently prioritized, or operationalized locally. This resonates with research showing that policy ambitions frequently lose specificity and are reshaped as they are absorbed into broader health and social care reforms (Czarniawska and Sevón, 2005; Polacsek et al., 2020; Elg et al., 2025). The findings also show that these interpretive difficulties were tied to organizational conditions. Time pressure, workforce instability, documentation demands, and limited continuity did not simply obstruct person-centered care from the outside; they shaped what staff could reasonably understand person-centered care to mean in daily work. This supports prior research showing that person-centered dementia care depends not only on staff attitudes or skills, but on whether care environments allow relational work, flexibility, and professional judgment (Narayanasamy and Penney, 2014; Goh et al., 2022; Polacsek et al., 2020; Xu et al., 2024).

Our findings further suggest that tools such as the Life Story do not become meaningful simply because they align with person-centered ideals. Rather, their use reflected wider tensions between documentation, relational care, continuity, and role clarity. Their enactment depends on whether their purpose is understood, whether they fit everyday workflows, and whether staff perceive them as useful in supporting care in the present. This supports work showing that the uptake of person-centered tools depends on their practical relevance and on how they interact with existing routines and professional judgments (McKeown et al., 2010; Brower, 2025; Xu et al., 2024).

A second contribution is the refinement of how variation in enactment is understood. Our findings suggest that in dementia homecare, some degree of variation is necessary because people's needs, communication, and capacity change over time (Hoel et al., 2021; Hedman et al., 2022). The problem arises when interpretation remains largely individualized and unsupported by coordination, feedback, and shared learning. In those conditions, variation may reflect uneven access to guidance, time, or organizational support rather than context-sensitive care. We therefore distinguish between productive adaptation, in which situated interpretation remains connected to broader policy goals, and stalled enactment, in which fragmentation prevents coordinated action across roles and settings.

Differences between managerial and frontline perspectives should not be read simply as misalignment. Rather, they reflect the distinct responsibilities of actors positioned differently within the homecare system. In this study, managers more often emphasized structural constraints, whereas frontline staff and unit managers foregrounded relational work and adaptation to changing needs. What appeared consequential was not difference itself, but the limited dialogue between these perspectives. Where such dialogue was weak, experiential knowledge from everyday care was less likely to shape organizational decisions.

A third contribution concerns the capacity to enact person-centered dementia care. In the study, this depended not only on dementia-related knowledge, but also on communication, relational care, ongoing needs assessment, and clearer role coordination across the system. This aligns with literature showing that person-centered dementia care requires relational, communicative, and interpretive capacities, especially where preferences and needs must be inferred, revisited, or negotiated over time (Brooker, 2007; Hedman et al., 2022; Xu et al., 2024). Our findings add that these competencies are weakened when work remains organized primarily around tasks and standardized documentation, responsibilities for relational work, follow-up, and decision-making remain unclear, and when homecare work is undervalued despite its increasing complexity. Training therefore remains necessary, but insufficient unless organizations also clarify roles and create conditions that allow these competencies to be used in practice.

A final contribution concerns the role of participatory design in supporting policy enactment. The workshops created structured spaces in which actors from different roles could compare interpretations, surface tensions, and reflect on how policy concepts and routines were being operationalized. This supports research suggesting that participatory and co-design approaches can aid collective sensemaking in complex care and public-sector settings (Kimbell and Bailey, 2017; Blomkamp, 2022; Einfeld and Blomkamp, 2022; Matos-Castaño et al., 2025). In our study, Workshop 1 showed that the policy idea of co-creation could be translated into multiple practice-oriented interpretations, while Workshop 2 showed that challenges surrounding the Life Story were tied to the wider organization of care rather than to the tool alone.

These findings support the usefulness of what we term middle-level interpretations: shared, provisional orientations that connect abstract policy ambitions with locally meaningful directions for action. Their value lies not in fixing one final meaning of policy, but in making alignment work possible across roles and contexts. At the same time, their influence on practice likely depends on sustained engagement, follow-up, and organizational support. Participatory design therefore functioned less as a stand-alone solution than as an exploratory and coordinating complement to leadership, continuity, practical guidance, and stronger feedback mechanisms. Overall, the study suggests that the enactment of person-centered dementia care in homecare depends less on policy design alone than on the conditions that enable shared interpretation and coordinated action. Policies may articulate strong person-centered ambitions, but their practical force depends on whether organizations create time, legitimacy, and support for collective interpretation in everyday care work.

### 5.1 Implications for dementia policy and practice

Our findings suggest that advancing person-centered dementia care in homecare requires moving beyond the introduction of tools or guidelines toward strengthening the organizational conditions that support their use in practice.

For policymakers, this means complementing policy formulation with mechanisms for interpretation across system levels, particularly in relation to dementia-specific care, which was often less visible within broader person-centered reforms. Moreover, our findings call for the integration of bottom-up perspectives, where insights from municipal practice can inform policy refinement. Clearer articulation of expectations and clearer delineation of roles across professional groups may support more consistent enactment. The findings also point to the need to address the economic and symbolic recognition of homecare work, which may affect workforce stability and the prioritization of relational care.

For municipal managers, the findings highlight the importance of leadership practices that support shared interpretation and coordination across roles. This includes creating regular opportunities for reflection, considering the voices of staff and people with lived experience, clarifying roles and responsibilities for person-centered care in dementia, and supporting continuity in both frontline and managerial roles. Recognizing and legitimizing relational work, and not only task completion, may help to reduce tensions between organizational demands and person-centered care.

For homecare organizations and teams, our findings suggest the need to adapt tools and routines to the temporal and relational conditions of dementia care. This may include integrating tools such as the Life Story into existing workflows, promoting spaces for reflection, clarifying how information should inform care and supporting staff in developing communication and relational skills.

### 5.2 Limitations and future research

This study has several limitations. First, it is situated in three municipalities in Sörmland, which may limit the transferability of findings to other regions and systems. Second, the workshops were subject to possible self-selection bias, as individuals with a particular interest in PCC or dementia care may have been more likely to take part, shaping the perspectives represented. Relatedly, background characteristics were captured more fully for interviewees than for workshop participants, reflecting the role of interviews as the primary dataset but limiting how fully the workshop sample can be described. Third, we only interviewed 17 individuals, which limits the extent to which the full complexity of enactment processes could be captured. Most participants belonged to the municipal level, meaning that some perspectives, particularly those of regional and national policymakers, were less represented.

Finally, the exploratory and time-bounded design of this study does not allow conclusions about the effectiveness or long-term sustainability of participatory, design-driven methods for policy enactment. Although the workshops created structured spaces for collective sensemaking and reflection, their limited duration and scope mean they could not capture how such processes evolve over time, interact with organizational routines, or shape later cycles of enactment. Future research would benefit from larger and more diverse samples, as well as longitudinal and comparative designs examining how collective sensemaking develops over time, whether middle-level interpretations shape longer-term enactment trajectories, and how participatory approaches interact with institutional power dynamics and organizational conditions in dementia care.

## 6 Conclusion

This study examined how person-centered dementia policies are enacted in municipal homecare and how participatory design approaches may support this process. The findings show that challenges in enactment are not driven by resistance to policy ideals, but by the difficulty of translating them into coordinated practice across roles, routines, and organizational conditions. First, the study highlights that enactment depends on alignment between abstract policy ambitions and the organizational realities of homecare. When policy meanings remain ambiguous or disconnected from everyday work, they are interpreted pragmatically and unevenly across roles. Second, we show that variation in practice is not inherently problematic, but becomes limiting when shared interpretation and coordination are lacking. We therefore refine the concept of policy stall by distinguishing between productive adaptation and stalled enactment resulting from persistent fragmentation. Third, the study demonstrates that participatory, design-driven approaches can support policy translation by creating structured spaces for collective sensemaking. Through these processes, actors can develop middle-level interpretations. Overall, the study suggests that improving the enactment of person-centered dementia care requires not only better policies or training, but stronger support for shared interpretation, coordination, and reflection within everyday care work.

## Data Availability

Due to ethical considerations and participant confidentiality, raw interview data are not available for sharing. Pseudonymized excerpts and selected aggregated materials may be provided upon reasonable request, where this does not conflict with ethical approvals or informed consent. Requests to access the datasets should be directed to Andrea Nakakawa, andrea.nakakawa@polimi.it.
