# Supplementary material for: Translating person-centered care into homecare practice: challenges and innovations in implementing dementia policy in Sweden
Source: Front Dement. 2026 May 15;5:1814577. doi: 10.3389/frdem.2026.1814577 (PMC13218968; doi:10.3389/frdem.2026.1814577)
Supplement: Supplementary file 1 [file Table_1.docx]

Translating Person-Centered Care into Homecare Practice: Challenges and Innovations in Implementing Dementia Policy in Sweden

Andrea Nakakawa^1^, Jenny Löyttynen^2^, Daniela Sangiorgi^1^, Lena Marmstål Hammar^2*^

Supplementary Materials

**Annex 1.** Overview of interview guide

| **Section** | **Objective** | **Estimated time** | **Example questions** |
| --- | --- | --- | --- |
| Introduction | Provide an overview of how the interview will be conducted, reassure confidentiality, and answer any questions | 5 min | Do you have any questions before we start?  Do you consent to having this interview recorded? |
| Person-centricity (key values) in dementia care   - Definition and understanding - Policies | Learning about what do participants understand as person-centered care and key values around it.  Understand the level of familiarity of participants with national and local policies connected to dementia and person-centered care | 15 min | What is person-centered care?  What do you understand by autonomy / personhood / citizenship?  Are you aware of any policy documentation that is relevant when talking about these values? |
| Operationalization of PCC policies | Understand the process of translating national and local policies into action plans, routines, guidelines, and behaviors | 15 min | How are these protocols, routines or “way of doing things” being transmitted to others?  Are there opportunities for you to provide feedback to existing policies / guidelines for homecare provision? |
| Challenges and opportunities | Identify challenges and opportunities in the operationalization of PCC policies in the context of dementia | 20 min | What are the main challenges you face to provide PCC to people with dementia in your everyday activities?  What resources, conditions, or support would enable you to better promote the autonomy, personhood, and citizenship of people with dementia living at home? |
| Closing | Capture any final comments and thank the participant for their time | 5 min |  |

**Annex 2.** Overview of the two workshops’ structure

| **Workshop 1** | | |
| --- | --- | --- |
| **Phase** | **Objective** | **Activity** |
| Relating to the policy vision | Reflect on participants’ own ideas, values, and expectations related to co-creation of care | Presentation of a selected policy extract. Individual written reflection on what “being a co-creator of care” means, including keywords and examples of when participants would feel they are co-creating care. |
| Multiple perspectives mapping: Frame the policy in the context of dementia care | Share and contrast perspectives on co-creation in the context of dementia | Presentation of perspectives from people with dementia and informal carers (facilitator-led, supported by printed quotes), followed by guided small-group discussion on how co-creation is complicated by cognitive decline, relational decision-making, and care responsibilities. |
| Identifying existing practices + ideating | Identify current practices and opportunities that support co-creation in dementia homecare | Group brainstorming of existing practices that support co-creation, followed by scenario- and persona-based discussion to explore practices beyond participants’ immediate experiences. |
| Reframing the policy (create a middle-level interpretation) | Identify key attributes that could orient co-creation of care in practice | Groups reflected on the outputs of the ideation activity to extract shared attributes considered necessary to support co-creation of care for people with dementia. Attributes were discussed as provisional and practice-oriented rather than formal policy statements. |

| **Workshop 2** | | |
| --- | --- | --- |
| **Phase** | **Objective** | **Activity** |
| The policy visions behind the Life Story tool (LS) | Explore the policy visions and values underpinning the Life Story tool | Facilitated discussion around selected policy extracts related to the Life Story, person-centered care, and participation (e.g., Roadmap to Good and Close Care, National and Regional Dementia Strategies). Participants worked in small groups to identify key policy messages, underlying values, and expectations regarding participation, personalization, and relational care. |
| Understand and frame the challenge | Share experiences and surface challenges related to the use (or non-use) of the Life Story tool | 1. Presentation of interview findings related to the Life Story tool, focusing on reported challenges, uncertainties, and variations in use. 2. Small-group discussion where participants shared their own experiences of using the Life Story, including perceived purpose, practical difficulties, organizational constraints, and differences in how the tool is used, accessed, or consulted across roles and teams. |
| Prototyping | Ideate practice-informed alternatives or adaptations that could better align the Life Story with policy intentions | Collaborative group work using prototyping materials to explore alternative formats, workflows, and supporting arrangements for the Life Story. Rather than redesigning the template alone, participants considered broader socio-material configurations, including roles and responsibilities, timing, skills, continuity, documentation practices, and the use of digital or sensory supports. |
| Middle-level interpretation | Identify key attributes emerging from the prototypes that could orient the enactment of the Life Story in practice | Groups reflected on their proposed prototypes to articulate shared attributes and conditions considered necessary for the Life Story to function meaningfully in dementia homecare (e.g., adaptability across dementia stages, relational continuity, clarity of purpose, shared access across professional roles). These attributes were discussed as provisional orientations rather than finalized solutions. |
